# Maize *Dek407* Encodes the Nitrate Transporter 1.5 and Is Required for Kernel Development

**DOI:** 10.3390/ijms242417471

**Published:** 2023-12-14

**Authors:** Hongqiu Wang, Xiaolan Yan, Qingguo Du, Pengshuai Yan, Jinjin Xi, Xiaoruo Meng, Xuguang Li, Huijian Liu, Guoqin Liu, Zhongjun Fu, Jihua Tang, Wen-Xue Li

**Affiliations:** 1 National Engineering Laboratory for Crop Molecular Breeding, Institute of Crop Sciences, Chinese Academy of Agricultural Sciences, Beijing 100081, China; 2 National Key Laboratory of Wheat and Maize Crop Science, College of Agronomy, Henan Agricultural University, Zhengzhou 450002, China; 3 The Shennong Laboratory, Zhengzhou 450002, China; 4 Chongqing Academy of Agricultural Sciences, Chongqing 401329, China

**Keywords:** maize, defective kernel, IAA, kernel size, kernel weight, natural variation

## Abstract

The kernel serves as the storage organ and harvestable component of maize, and it plays a crucial role in determining crop yield and quality. Understanding the molecular and genetic mechanisms of kernel development is of considerable importance for maize production. In this study, we obtained a mutant, which we designated defective kernel 407 (*dek407*), through ethyl methanesulfonate mutagenesis. The *dek407* mutant exhibited reduced kernel size and kernel weight, as well as delayed grain filling compared with those of the wild type. Positional cloning and an allelism test revealed that *Dek407* encodes a nitrate transporter 1/peptide transporter family (NPF) protein and is the allele of *miniature 2* (*mn2*) that was responsible for a poorly filled defective kernel phenotype. A transcriptome analysis of the developing kernels showed that the mutation of *Dek407* altered the expression of phytohormone-related genes, especially those genes associated with indole-3-acetic acid synthesis and signaling. Phytohormone measurements and analysis indicated that the endogenous indole-3-acetic acid content was significantly reduced by 66% in the *dek407* kernels, which may be the primary cause of the defective phenotype. We further demonstrated that natural variation in *Dek407* is associated with kernel weight and kernel size. Therefore, *Dek407* is a potential target gene for improvement of maize yield.

## 1. Introduction

Maize is among the most important food crops worldwide, as well as an important feed source and bioenergy crop, thereby playing a pivotal role in addressing global food insecurity and facilitating the production of clean energy [1]. Progress on these issues can be effectively achieved through the improvement of maize yield. Maize yield is a complex trait that encompasses multiple secondary traits, but it is primarily determined by the number of ears per unit area, the number of kernels per ear, and the weight of the kernels. Maize kernel development directly affects the kernel weight. Thus, the identification and cloning of key regulatory genes involved in maize kernel development is instrumental in the genetic enhancement of maize yield.

To date, innumerable maize mutants that are defective in kernel development have been identified. These mutants can be categorized into a variety of kernel mutant types based on their mutant phenotypes. For example, the defective kernel (*dek*) mutants are characterized by impaired development of both the embryo and endosperm [2]. The empty pericarp (*emp*) mutants exhibit either an empty pericarp or a papery kernel [3]. The embryo-specific (*emb*) mutants display morphogenic effects that are specific to the embryo [4]. Numerous genes involved in maize kernel development have been identified through the characterization of these mutants. For example, the genes *Dek605*, *Ppr2263*, *Smk1*, *PCW1*, *Emp8*, and *Emp10* encode PLS- or P-type pentatricopeptide repeat (PPR) proteins that regulate maize embryo and endosperm development by participating in mitochondrial RNA intron splicing or site-specific editing [5,6,7,8,9,10]. *Smk2* encodes a glutaminase that regulates maize seed development by influencing the biosynthesis of vitamin B_6_ [11]. *Mn1* and *Meg1* encode a cell wall sucrose invertase and cysteine-rich polypeptide, respectively, and their mutation affects the development of cells in the basal endosperm transfer layer (BETL) [12,13]. *Pro1* encodes a carboxylate synthase that influences kernel development by regulating proline synthesis and GCN2 (general control nonderepressible 2) kinase activity [14]. *ZmUrb2*, *ZmShrek1*, *Reas1*, and *RCL1* encode diverse types of ribosome biosynthesis factors that participate in ribosome biogenesis during kernel development [15,16,17,18].

Nitrogen is a vital element for plant growth, metabolism, and heredity, and its nutritional status directly impacts crop yield. Nitrate is an abundant source of nitrogen in the soil and the primary form of nitrogen absorbed by plants [19,20]. The nitrate transporters (NRTs) play extensive, crucial roles in the uptake of nitrate from the soil and its subsequent translocation to various plant organs, thereby facilitating plant growth and development. In the past 30 years, four families of transporters involved in nitrate transport have been identified in plants, comprising the nitrate transporter 1 (NRT1)/peptide transporter (PTR) family (NPF), NRT2 family, chloride channel (CLC) family, and slowly activating anion channel (SLAC) family [21,22]. Their functions have been extensively described elsewhere [22,23]. 

The *NPF* gene family of *Arabidopsis thaliana* and rice (*Oryza sativa*) comprises 53 and 93 gene members, respectively, whereas in maize, the *NPF* gene family consists of 78 genes [23,24,25]. In higher plants, all members of the NPF protein family have 12 conserved transmembrane domains (TMs), with a large hydrophilic loop located between TM6 and TM7 [26]. The NPF family proteins, like AtNPF2.3, AtNPF6.3, AtNPF7.2, OsNPF2.2, and OsNPF2.4, were initially identified as nitrate or peptide transporters [27,28,29,30,31]. However, in recent years, more and more NPF proteins have been shown to transport a variety of substrates, ions including Cl^−^ and K^+^, amino acids, sucrose, glucose, and hormones including indole-3-butyric acid (IBA), indole-3-acetic acid (IAA), abscisic acid (ABA), jasmonates (JAs), and gibberellins (GAs) [32,33,34,35]. In *Arabidopsis*, AtNPF2.4 and AtNPF2.5 have been reported to exhibit chloride transport capacity [36,37]. Recent research revealed that the AtNPF7.3 transporter exhibits not only nitrate and K^+^ transport activities, but also the capability to transport IBA, which serves as a precursor in the synthesis of IAA [34,38]. The AtNPF7.3/NRT1.5 transporter may regulate root geotropism by mediating IBA uptake in root cells and maintaining an optimal auxin gradient within the root [34]. The ability of AtNPF3.1 and AtNPF4.6 to transport ABA was confirmed using a functional expression system in *Xenopus laevis* [39,40]. The AtNPF3 transporter is capable of efficiently transporting GA in vitro and in vivo, thereby affecting GA accumulation in the root endoderm [39]. Recent study in maize found that a NRT1/PTR-type transporter ZmSUGCAR1, the homologous protein of AtNPF7.3, also exhibits the additional capacity to transport sucrose, glucose, and K^+^, but not IAA [35]. The diversity of transport substrates of NPF transporters indicates that NPF transporters may perform extensive functions in plant growth and development [32].

To date, the mechanism by which NPF proteins regulate maize kernel development remains largely unknown. In the present study, we obtained the defective kernel 407 (*dek407*) mutant through ethyl methanesulfonate (EMS) mutagenesis. Using bulked segregant RNA-sequencing (BSR-seq) analysis, positional cloning, and an allelism test, we identified *Dek407* as a variant of *Mn2*/*ZmNPF7.9*/*ZmSUGCAR1* that was responsible for the poorly filled defective kernel phenotype and proposed to be a sucrose transporter [35,41,42]. *Dek407* encodes an NPF protein with 12 conserved TMs. Our further analysis in the *dek407* mutant showed that the G/A mutation in the third TM altered the expression of phytohormone-related genes and the phytohormone balance, especially the expression of IAA-related genes and the content of IAA. Finally, we showed that natural variation in *Dek407* is associated with kernel weight and kernel size. This study provides a foundation for further elucidating the function of NPF protein involved in maize kernel development and hormone transport. 

## 2. Results

### 2.1. Phenotypic and Genetic Analysis of the dek407 Mutant

**Figure 1:**
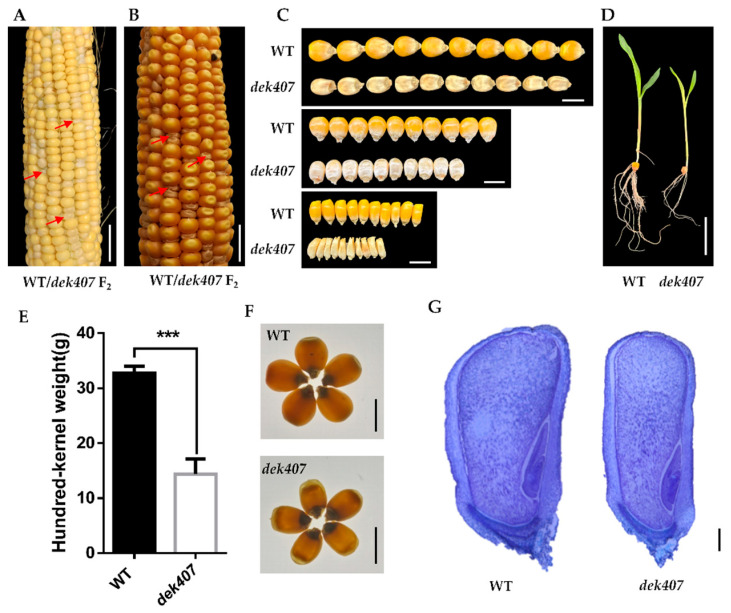
Phenotypic features of the maize *dek407* mutant. (**A**) Self-pollinated *dek407* heterozygote ears at 12 days after pollination (DAP). The red arrows indicate the *dek407* mutant kernels. Bar = 2 cm. (**B**) Mature *dek407* heterozygote ears. The red arrows indicate the *dek407* mutant kernels. Bar = 2 cm. (**C**) The kernel length, kernel width, and kernel thickness of WT and *dek407* mature kernels, which were randomly selected from the heterozygote ears. Bar = 1 cm. (**D**) Phenotypes of WT and *dek407* seedlings at 10 days after sowing (DAS). Bar = 5 cm. (**E**) Hundred-kernel weight of the WT and *dek407* mutant. Error bars indicate SE. ***, *p* < 0.001 (*t*-test), significant difference from the WT. (**F**) WT and *dek407* kernels viewed on a light box. Bar = 1 cm. (**G**) Micrograph of longitudinal sections of WT and *dek407* kernels stained with toluidine blue at 12 DAP. Bar = 1 mm.

To elucidate the molecular regulatory mechanisms of maize kernel development, we screened an EMS-mutagenized library in the maize B73 genetic background for mutants with kernel development defects [43]. One mutant, which we designated defective kernel 407 (*dek407*), showed severely defective kernel development. The mutant seeds were easily identified as having a reduced kernel size and delayed grain filling in the heterozygous ears at 12 days after pollination (DAP) (Figure 1A). The *dek407* mature kernels exhibited a small, wrinkled kernel phenotype together with an empty pericarp at the top (Figure 1B,C and Figure S1D). Compared with wild-type (WT) kernels, the kernel length, width, thickness, and hundred-kernel weight of the *dek407* mutant were significantly reduced by 6%, 21%, 36%, and 56%, respectively (Figure 1C and Figure S1A–C). When observed on a light box, the *dek407* kernels exhibited greater light transmittance than the WT kernels (Figure 1F). Longitudinal sections of *dek407* mature kernels revealed a much less relative floury endosperm area (Figure S1D,E). Paraffin-embedded sections of the developing kernels showed that the embryo in the mutant was reduced in size compared to the WT (Figure 1G), along with a small embryo in the mature *dek407* kernels (Figure S1D). The *dek407* seedlings showed a decrease in plant height, root length, and root number at 10 days after sowing (DAS) (Figure 1D). In addition, the starch content in the *dek407* mutant kernels was 24% lower than that in the WT kernels (Figure S1E).

**Figure 2:**
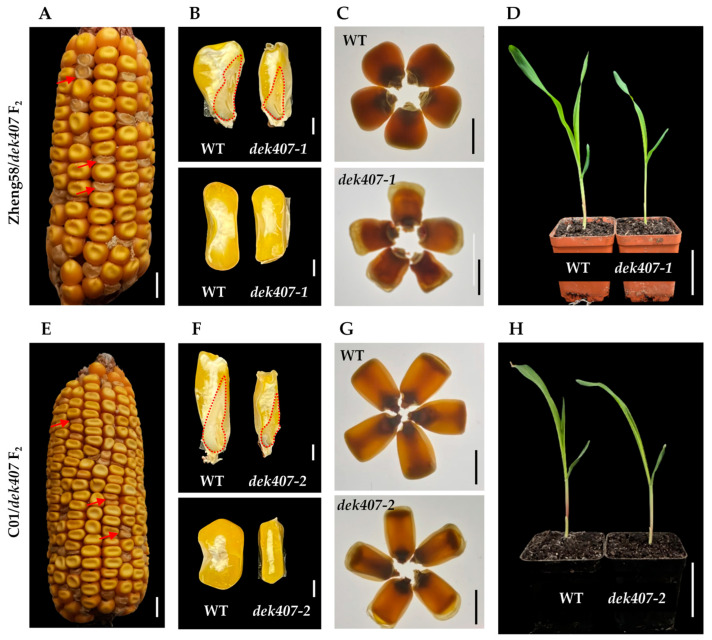
Phenotypic features of maize *dek407* mutants in Zheng58 (*dek407-1*) and C01 (*dek407-2*) backgrounds. (**A**) Mature F_2_ ear of *dek407* × Zheng58. The red arrows indicate the mutant kernels (*dek407-1*). Bar = 1 cm. (**B**) Longitudinal and transection sections of WT and *dek407-1* kernels. The part circled by the red dotted line represents the embryo. Bar = 2 mm. (**C**) WT and *dek407-1* kernels viewed on a light box. Bar = 1 cm. (**D**) Phenotypes of WT and *dek407-1* seedlings at 10 DAS. Bar = 5 cm. (**E**) Mature F_2_ ear of *dek407* × C01. The red arrows indicate the mutant kernels (*dek407-2*). Bar = 1 cm. (**F**) Longitudinal and transection sections of WT and *dek407-2* kernels. The part circled by the red dotted line represents the embryo. Bar = 2 mm. (**G**) WT and *dek407-2* kernels viewed on a light box. Bar = 1 cm. (**H**) Phenotypes of WT and *dek407-2* seedlings at 10 DAS. Bar = 5 cm.

To further analyze the phenotype and segregation of *dek407* in different genetic backgrounds, the *dek407* homozygote was crossed with the Zheng58 and C01 inbred lines to generate two F_2_ populations. The mutant kernels in the two populations, *dek407-1* (in the Zheng58 background) and *dek407-2* (in the C01 background), also exhibited a small, wrinkled kernel phenotype, together with an empty pericarp at the top (Figure 2A–C,E–G). The kernel width, thickness, and hundred-kernel weight of *dek407-1* were reduced by 20%, 33%, and 49%, respectively, compared with those of the WT, whereas the kernel length showed no significant difference (Figure S2A–E). In *dek407-2*, the kernel length, width, thickness, and hundred-kernel weight were reduced by 7%, 11%, 25%, and 45%, respectively, compared with those of the WT (Figure S2F–J). At 10 DAS, the seedling development of *dek407-1* and *dek407-2* was delayed compared with the WT seedlings (Figure 2D,H). The segregation ratio of WT kernels to mutant kernels in each of the two F_2_ populations was consistent with a 3:1 Mendelian segregation ratio (Table S1), suggesting that *dek407* is a recessive mutation under monogenic control. All of the aforementioned results indicated that kernel development and seed size were affected in the *dek407* mutant. 

### 2.2. Positional Cloning and Identification of the Dek407 Gene

**Figure 3:**
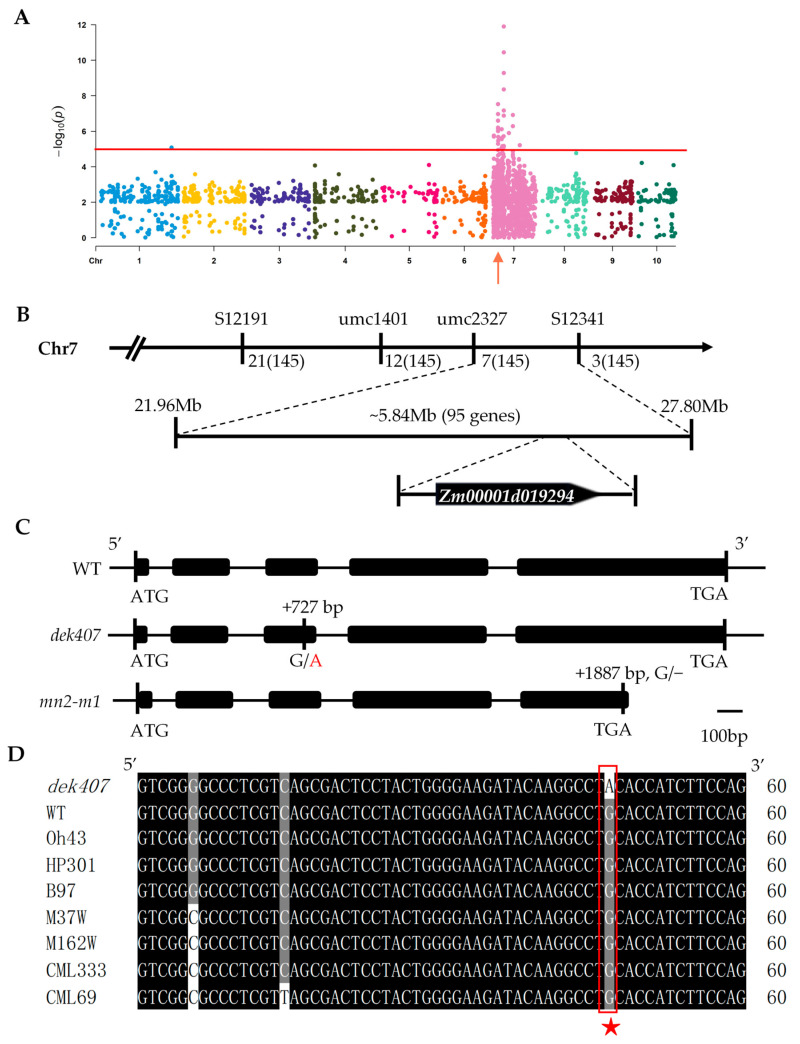
Cloning and identification of *Dek407*. (**A**) Frequency variance analysis result of bulk segregant RNA-seq (BSR-seq). The 10 chromosomes are represented with different colors, and the candidate region is indicated by a red arrow. The red line indicates the threshold. (**B**) The *dek407* locus was mapped to a 5.84 Mb region on chromosome 7 between the SSR marker umc2337 and the InDel marker S12341. (**C**) Schematic diagram of the *dek407* and *mn2-m1* coding regions. Black boxes indicate exons. (**D**) Analysis of *dek407* mutant site in other maize inbred lines. The red rectangle indicates the mutant site in other maize inbred lines. Red star indicates mutant site in *dek407*.

To preliminarily determine the chromosomal location of *Dek407*, we conducted bulked segregant RNA-sequencing (BSR-seq) using kernels from the homozygous *dek407* mutant and WT kernels from segregated F_2_ ears at 15 DAP. The endosperm RNA isolated from 30 WT and 30 mutant seeds was mixed equally to generate two pools of extreme bulked segregants for BSR-seq analysis. We sequenced the two extreme bulked pools on an Illumina platform using 150 bp paired-end reads to obtained the polymorphic SNP markers. The genotype frequencies (SNP-index) of each polymorphic SNP marker were calculated after removing the non-polymorphic and low-quality marker. A 32.52 Mb region (22.12 Mb to 54.64 Mb) on chromosome 7 may be the causal locus for *dek407* based on the occurrence of significant linkage disequilibrium (Figure 3A). 

To further confirm and narrow the candidate interval, two insertion/deletion (InDel) markers (S12191 and S12341) and two simple sequence repeat (SSR) markers (umc1401 and umc2327) were developed to identify the genotype of the *dek407-2* F_2_ population (145 mutant kernels). The *Dek407* gene was mapped to a 5.84 Mb region containing 95 annotated genes based on the B73-REFERENCE-GRAMENE-4.0 genome assembly (https://www.maizegdb.org/genome/assembly/Zm-B73-REFERENCE-GRAMENE-4.0) (accessed on 1 January 2022) (Figure 3B). Among the 95 annotated genes, we observed that *Zm00001d019294* encodes the previously reported nitrate transporter 1/peptide transporter family protein, which is responsible for the small kernel phenotype of *mn2* [41]. We sequenced the full-length nucleotide sequence of *Zm00001d019294* in the homozygous WT and mutant, which revealed the presence of a non-synonymous mutation (G/A) in the third exon (+727 bp from ATG) in *dek407*, leading to an alteration of the corresponding coding amino acid from cysteine (Cys) to tyrosine (Tyr) (Figure 3C). We also analyzed the specificity of this mutation site in other maize inbred lines and determined that only *dek407* contained this mutation site (Figure 3D). These results suggested that the G/A mutation in the third exon of *Zm00001d019294* may be responsible for the mutant phenotype. 

**Figure 4:**
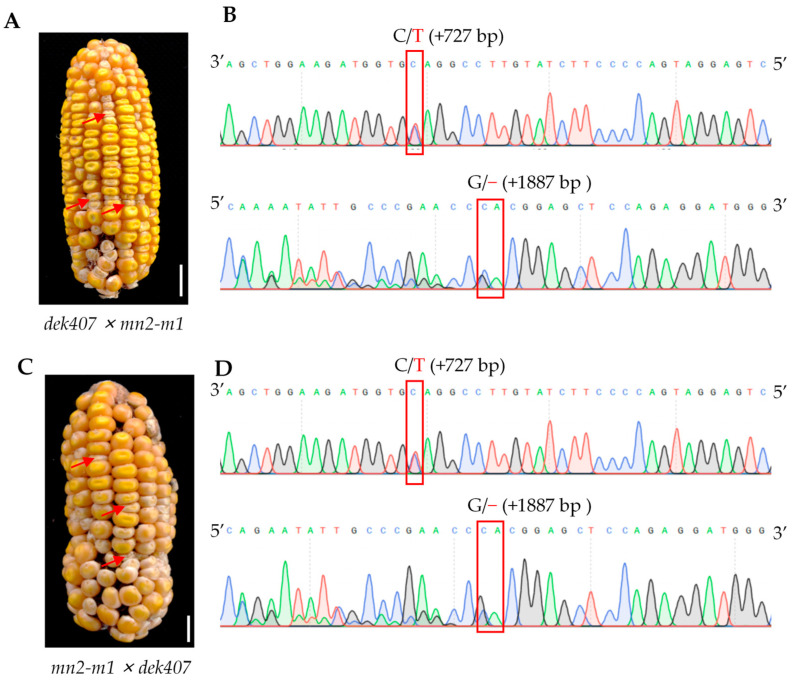
Allelism test of *dek407* with *mn2-m1*. (**A**) Allelism test ear of heterozygous *dek407* × *mn2-m1*. The red arrows indicate the mutant kernels. Bar = 1 cm. (**B**) Sequence analysis of the mutant kernels from the allelism test ear. The locations of the *dek407* and *mn2-m1* mutant sites are shown in the red box. C/T indicates the mutant site in *dek407* and G/− indicates the mutant site in *mn2-m1*. (**C**) Allelism test ear of heterozygous *mn2-m1* × *dek407*. The red arrows indicate the mutant kernels. Bar = 1 cm. (**D**) Sequence analysis of the mutant kernels from the allelism test ear. The locations of the *dek407* and *mn2-m1* mutant sites are shown in the red box.

To validate *Zm00001d019294* as the allele of *dek407*, we crossed the heterozygous plant *mn2-m1 (+/−)* with *dek407 (+/−)* for an allelism test. The *mn2-m1* mutant had a G/− deletion in the fifth exon of *Zm00001d019294*, resulting in premature termination of protein translation (Figure 3C) [41]. The allelism test in two separate ears revealed a 3:1 segregation ratio of WT to mutant kernel phenotypes, with 269 (or 146) WT and 89 (or 41) mutant kernels (Figure 4A,C and Table S2). Mutant kernels from the two ears were randomly selected for genotyping to verify the allelism test results (Figure 4B,D). These results confirmed that *Zm00001d019294* was indeed the *Dek407* gene.

### 2.3. Dek407 Encodes a Highly Conserved Nitrate Transporter Protein and Is Predominantly Expressed in Developing Seeds

**Figure 5:**
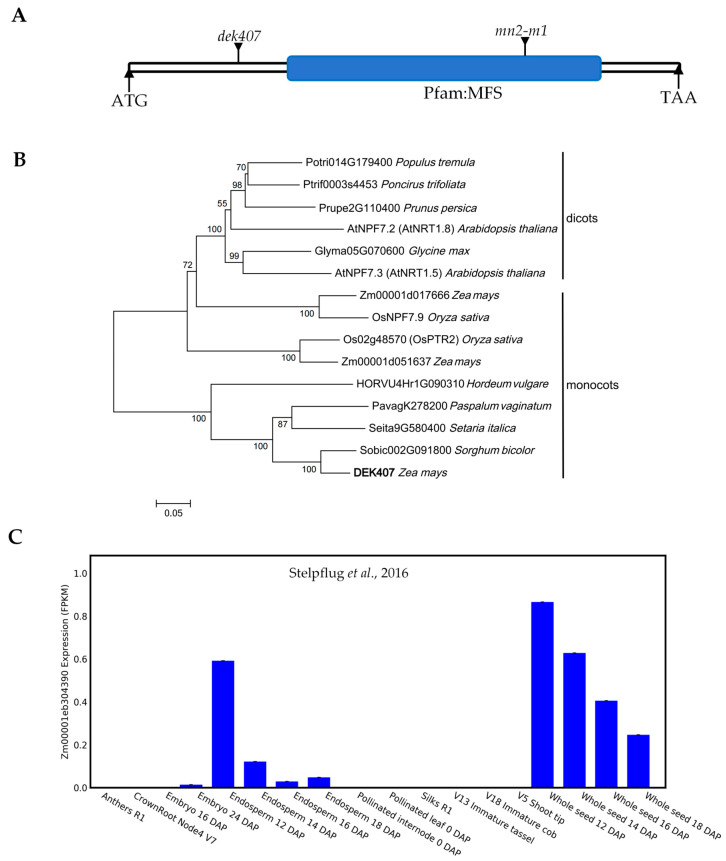
Phylogenetic relationship and expression pattern of DEK407. (**A**) The predicted major facilitator superfamily (MFS) domain of the DEK407 protein. (**B**) Phylogenetic tree of DEK407 and its homologs in different species. The bold represents the DEK407 protein in maize. (**C**) Expression levels of *Dek407* in different maize organs from *qTeller* platform [44].

*Zm00001d019294* was previously annotated as *ZmNRT1.5/ZmNPF7.9* of the *NPF* transporter family [41,42]. A sequence analysis and protein domain prediction of *Zm00001d019294* based on *TOPCONS* (https://topcons.net) (accessed on 1 March 2022) and *SMART* (http://smart.embl-heidelberg.de) (accessed on 1 March 2022) revealed that it contains a 1923 bp open reading frame (ORF) that encodes a typical transmembrane protein containing 12 transmembrane domains (TMs) with a major facilitator superfamily (MFS) domain in the C terminal (Figure 5A and Figure S3). A phylogenetic tree was constructed based on the full-length DEK407 amino acid sequence and its homologs in 10 representative plant species. The homologs were resolved into monocotyledonous and dicotyledonous evolutionary groups (Figure 5B). DEK407 was most closely related to Sobic002G091800 from *Sorghum bicolor*, and it also exhibited a high amino acid sequence similarity with OsNPF7.9 and OsPTR2 of rice and with AtNPF7.3 and AtNPF7.2 from *Arabidopsis*. In maize, Zm00001d017666 and Zm00001d051637 shared a high sequence similarity with DEK407, but their most closely related homologs in rice were OsNPF7.9 and OsPTR2, respectively (Figure 5B). Detailed DEK407 amino acid sequence alignment with Zm00001d017666, Zm00001d051637, OsNPF7.9, OsPTR2, AtNPF7.3, AtNPF7.2, and Sobic002G091800 indicated that all of these homologs contained 12 conserved TMs, and the mutation site Cys/Tyr (C/Y) in *dek407* was located in the TM3 region (Figure S3).

The N-terminus of the DEK407 protein contained an NRT/PTR domain typical of the NPF family. Thus, we further analyzed the evolutionary relationships of 30 and 50 well-known NPF family proteins in rice and *Arabidopsis*, as well as their homologs in maize. The phylogenetic tree revealed that these NPF family proteins in maize were grouped into seven NPF subfamilies based on their homology with proteins in rice and *Arabidopsis* (Figure S4). DEK407 was classified into the NPF7 subfamily and belonged to a relatively distinct subbranch compared with other NPF7 members. Zm00001d017666 and Zm00001d051637 were classified to the same subbranch with OsNPF7.9 and OsPTR2, respectively (Figure S4).

As Zm00001d017666 and Zm00001d051637 shared a high sequence similarity with DEK407, we analyzed their spatiotemporal expression patterns based on the datasets accessible from the *qTeller* platform (https://qteller.maizegdb.org) (accessed on 1 March 2022) [44]. The results revealed that *Dek407* was predominantly expressed in developing seeds (Figure 5C). *Zm00001d017666* exhibited high expression levels in the internode, leaf, and taproot, but it displayed minimal expression in the seed (Figure S5A). *Zm00001d051637* was expressed specifically in the embryo (Figure S5B). Thus, these three homologs in maize potentially perform similar functions in different tissues owing to their tissue-specific expression. 

### 2.4. Comparative Transcriptome Analysis of Developing Kernels of the dek407 Mutant and WT

**Figure 6:**
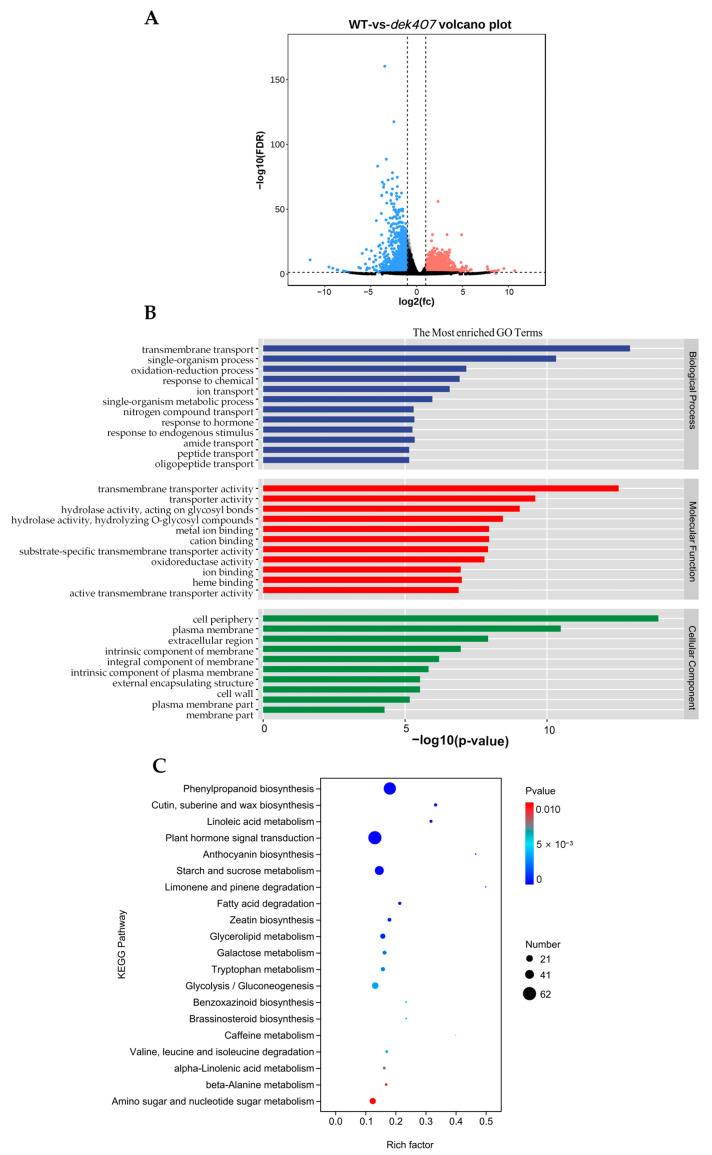
GO and KEGG pathway enrichment analysis of DEGs between WT and *dek407*. (**A**) Volcano plot used to visualize RNA-seq data. Each point corresponds to a DEG. Red and blue dots represent up-regulated and down-regulated genes in *dek407*, respectively. Black dots represent non DEGs. (**B**) Enriched GO terms of the DEGs. (**C**) Enriched KEGG pathways of the DEGs. The size of the circles denotes the number of genes, and the color of the circles denotes the range of the *p*-value.

To investigate the impact of *dek407* on kernel development at the whole-genome transcriptional level, we conducted a transcriptome analysis using total RNA isolated from WT and *dek407* mutant kernels at 15 DAP, with three biological replicates. Total transcripts of 25,769 and 27,078 genes were detected in the WT and *dek407* mutant, respectively, with fragments per kilobase of transcript per million mapped reads values higher than 0.1. Based on the criteria of a fold change (FC) > 2, fragments per kilobase million (FPKM) > 1, and false discovery rate (FDR) < 0.05, 3265 genes were identified as differentially expressed genes (DEGs). Among these genes, 2335 genes (72% of the 3265 DEGs) exhibited significantly increased transcript abundance in the *dek407* mutant, and 930 genes (28% of the 3265 DEGs) showed significantly decreased transcript abundance (Figure 6A).

To analyze the functional annotations and classifications of the DEGs, we performed a gene ontology (GO) enrichment analysis using all the down- and up-regulated DEGs. The DEGs were categorized into the three main GO categories, namely biological processes (BPs), molecular functions (MFs), and cellular components (CCs) (Figure 6B). The most highly enriched GO terms classified in the BP category were involved in transmembrane transport (GO:0055085, *p* = 1.20 × 10^−13^), ion transport (GO:0006811, *p* = 2.70 × 10^−7^), nitrogen compound transport (GO:0071705, *p* = 5.00 × 10^−6^), oxidation–reduction process (GO:0055114, *p* = 7.00 × 10^−8^), and response to hormone (GO:0009725, *p* = 4.70 × 10^−6^). The GO terms enriched in the MF category mainly included transmembrane transporter activity (GO:0022857, *p* = 3.00 × 10^−13^), ion binding (GO:0043167, *p* = 1.10 × 10^−7^), oxidoreductase activity (GO:0016491, *p* = 1.60 × 10^−8^), and hydrolase activity, acting on glycosyl bonds (GO:0016798, *p* = 9.20 × 10^−10^). The enriched CC category mainly included the plasma membrane (GO:0005886, *p* = 3.30 × 10^−11^), membrane part (GO:0044425, *p* = 5.30 × 10^−5^), cell periphery (GO:0071944, *p* = 1.20 × 10^−14^), and extracellular region (GO:0005576, *p* = 1.20 × 10^−8^) (Figure 6B). 

We also performed a Kyoto Encyclopedia of Genes and Genomes (KEGG) enrichment analysis to further explore the biological functions of the DEGs. Pathways with *p* < 0.05 were identified as significantly enriched pathways. The KEGG pathway analysis indicated that phenylpropanoid biosynthesis (zma00940, *p* = 1.62 × 10^−9^), plant hormone signal transduction (zma04075, *p* = 4.70 × 10^−5^), and starch and sucrose metabolism (zma00500, *p* = 7.80 × 10^−5^) were significantly enriched in the *dek407* mutant (Figure 6C). These above findings revealed that the expression of genes involved in transmembrane transport, the oxidation–reduction process, ion binding, plant hormone signal transduction, and starch and sucrose metabolism were significantly altered in the *dek407* mutant. Interestingly, the significantly enriched pathway involved in plant hormone signal transduction was not reported in *zmsugcar1-1* [35].

### 2.5. Mutation of Dek407 Alters the Expression of Genes Associated with Phytohormones

**Figure 7:**
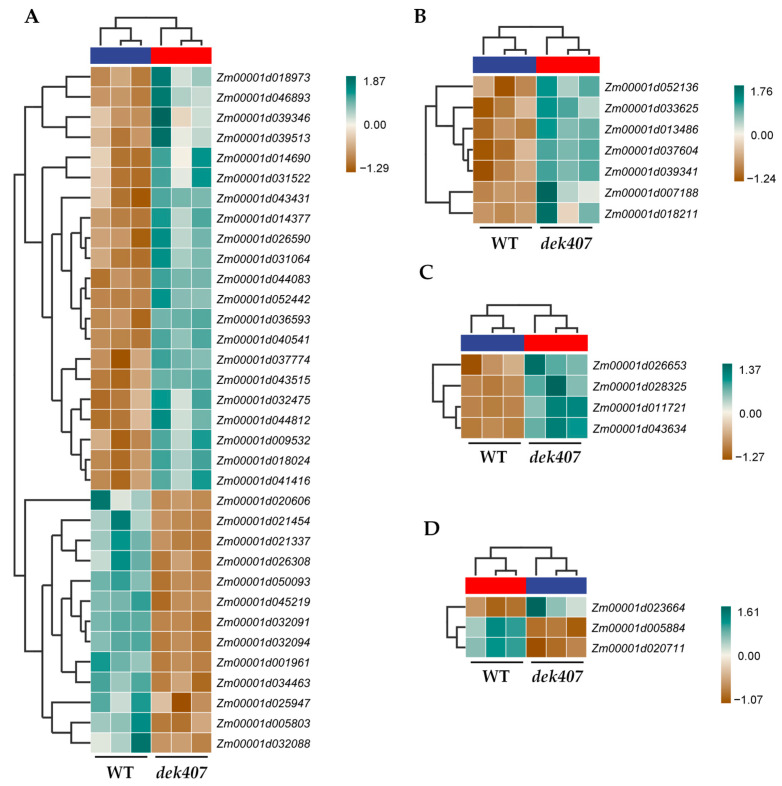
Heat map of plant hormone-related DEGs in WT and *dek407*. (**A**) IAA synthesis and signaling related DEGs. (**B**) Ethylene synthesis and signaling related DEGs. (**C**) BR synthesis and signaling related DEGs. (**D**) ABA signaling related DEGs. The red and blue box at the top of the heat map represent the three repetitions of the WT and *dek407*, respectively. The colors denote the range of the Z-Score.

Phytohormones, such as auxin, ethylene, ABA, and brassinosteroid (BR), play crucial roles in the development and maturation of plant seeds [45,46,47]. In the present study, the DEGs associated with hormone responses were identified as a significantly enriched GO subcategory (Figure 6B). In the KEGG enrichment analysis, the plant hormone signal transduction pathway was also identified as a significantly enriched pathway (Figure 6C). Therefore, we analyzed the expression of phytohormone-related DEGs in the *dek407* mutant. Of the 34 DEGs associated with IAA synthesis and signaling in the *dek407* mutant, seven DEGs encoding auxin response factor (ARF) family proteins and seven DEGs encoding auxin-responsive proteins were significantly up-regulated in the *dek407* mutant. Additionally, four DEGs encoding polar auxin transport (PIN) proteins were significantly up-regulated in the *dek407* mutant (except *PIN12*), and twelve DEGs encoding small auxin-up RNA (SAUR) family proteins were significantly down-regulated in the *dek407* mutant (except *SAUR56*) (Figure 7A and Table S3). All seven DEGs associated with ethylene synthesis and signaling, as well as four DEGs associated with BR synthesis and signaling, were significantly up-regulated in the *dek407* mutant (Figure 7B,C and Table S3). Three DEGs were involved in ABA signal transduction, comprising one up-regulated and two down-regulated genes (Figure 7D and Table S3). Altered expression of these genes may disturb hormone synthesis and signal transduction in the *dek407* mutant, leading to defects in kernel development.

### 2.6. The Phytohormone Balance Was Altered in the dek407 Mutant

**Figure 8:**
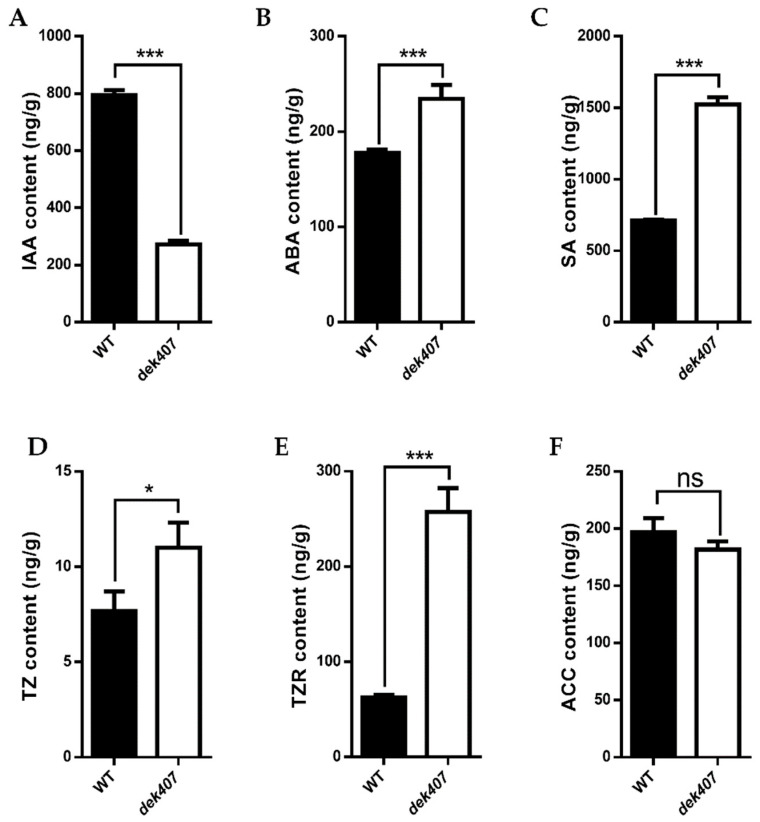
Measurement of endogenous hormones in WT and *dek407* kernels. (**A**) IAA (indole-3-acetic acid) content in WT and *dek407* kernels. (**B**) ABA (abscisic acid) content in WT and *dek407* kernels. (**C**) SA (salicylic acid) content in WT and *dek407* kernels. (**D**) TZ (trans-zeatin) content in WT and *dek407* kernels. (**E**) TZR (trans-zeatin riboside) content in WT and *dek407* kernels. (**F**) ACC (aminocyclopropane-1-carboxylic acid) content in WT and *dek407* kernels. Data are means ± SD (*** *p* < 0.001; * *p* < 0.05; n.s., not significant; Student’s *t*-test).

Auxin homeostasis has been reported to play indispensable roles in cereal endosperm development [48]. As the expression of genes associated with phytohormones was altered in the *dek407* mutant, we measured the contents of endogenous phytohormones in WT and *dek407* kernels using high-performance liquid chromatography–mass spectrometry (HPLC-MS). The results showed that the endogenous IAA content was significantly reduced by 66% in the *dek407* mutant (Figure 8A). The endogenous ABA and salicylic acid (SA) contents in the *dek407* mutant exhibited 1.32- and 2.14-fold increases, respectively, compared with those of the WT (Figure 8B,C). The contents of two important cytokinins, *trans*-zeatin (TZ) and *trans*-zeatin-riboside (TZR), in the *dek407* mutant exhibited 1.43- and 4.13-fold increases, respectively, compared with the WT (Figure 8D,E). The content of aminocyclopropane-1-carboxylic acid (ACC), the precursor of ethylene, exhibited no significant disparity between the WT and *dek407* mutant (Figure 8F). These results revealed that the phytohormone homeostasis was altered in the *dek407* mutant.

### 2.7. Natural Variations in Dek407 Are Associated with Kernel Weight and Kernel Size

**Figure 9:**
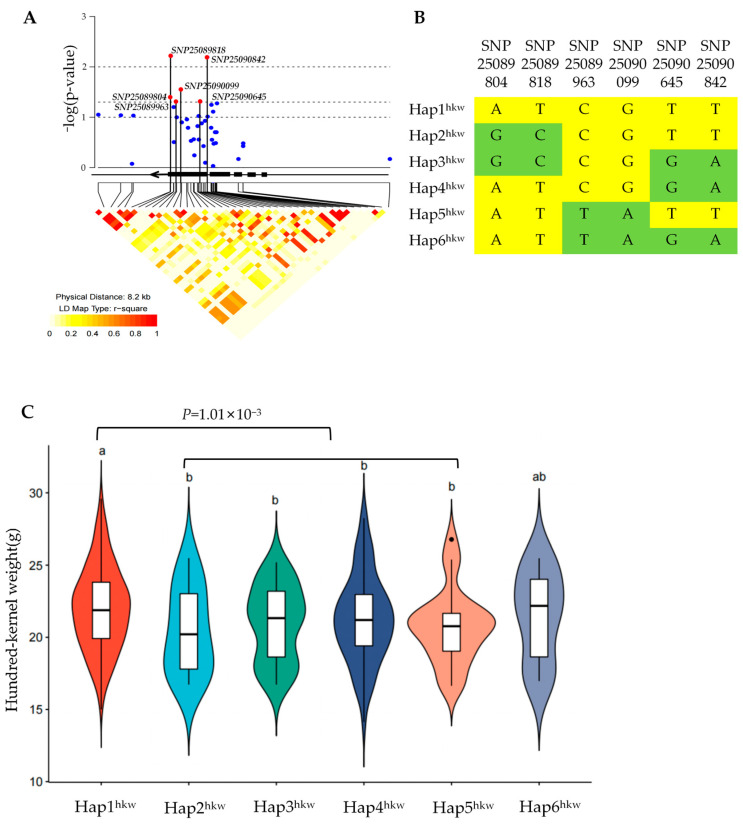
Natural variations in *Dek407* are significantly associated with maize hundred-kernel weight. (**A**) *Dek407*-based genetic variations associated with maize hundred-kernel weight and pairwise linkage disequilibrium (LD) analysis of the variations. Blue dots represent SNPs that are not significantly associated with hundred-kernel weight.(**B**) Haplotypes of *Dek407* hundred-kernel weight among these natural variations. Small letters ^hkw^ represents hundred-kernel weight. (**C**) Statistical analysis of hundred-kernel weight in the six haplotypes. Small letters ^hkw^ represents hundred-kernel weight. Means with the same small letter are not significantly different at *p* < 0.05 according to the LSD test.

To analyze the potential association between natural variations in the *Dek407* genomic region and traits associated with kernel weight and size, we performed a candidate gene association analysis using data from a panel of 503 maize genotypes. Six SNPs were observed to be significantly associated with kernel weight. Among these six SNPs, SNP25089804 and SNP25089818 were located in the 3′-untranslated region (3′-UTR), SNP25089963 (Ala/Thr), SNP25090099 (synonymous variant), and SNP25090645 (synonymous variant) were located in the fifth exon, and SNP25090842 was located in the fourth intron of *Dek407* (Figure 9A). The available 372 maize inbred lines were classified into six haplotypes (Hap) based on the six SNPs (Figure 9B). Hap2^hkw^, Hap3^hkw^, Hap4^hkw^, and Hap5^hkw^ exhibited no significant difference in their hundred-kernel weight (hkw), but all showed a significant reduction in their hundred-kernel weight compared with Hap1^hkw^ (Figure 9C). The Hap6^hkw^ exhibited no significant difference compared with any of the other haplotypes (Figure 9C). When Hap2^hkw^, Hap3^hkw^, Hap4^hkw^, and Hap5^hkw^ were grouped together, the merged group still exhibited a significantly reduced hundred-kernel weight compared with that of Hap1^hkw^ (Figure 9C). Therefore, Hap1^hkw^ of *Dek407* may be an excellent haplotype for kernel weight.

Three SNPs located in exon 4, SNP25090953 (Lys/Asn), SNP25090976 (Thr/Ala), and SNP25091118 (synonymous variant), were significantly associated with kernel thickness (kt) (Figure S6A). Based on these three SNPs, the available 448 maize inbreed lines were categorized into two haplotypes, Hap1^kt^ and Hap2^kt^ (Figure S6B). Hap2^kt^ was considered to be an excellent haplotype for kernel thickness owing to its significant increase in kernel thickness compared with that of Hap1^kt^ (Figure S6C). Eleven SNPs located in intron 2 (SNP25091853 and SNP25091855), exon 4 (SNP25090961 and SNP25091025), intron 4 (SNP25090842), and exon 5 (SNP25090099, SNP25090606, SNP25090645, SNP25090686, SNP25090738, and SNP25090788) were significantly associated with kernel width (kw) (Figure S7A). Among these SNPs, only SNP25090961 and SNP25090788 were missense variants, resulting in Gly/Ser and Phe/Leu mutations, respectively. Based on these 11 SNPs, the available 334 maize inbred lines were classified into seven haplotypes (Figure S7B). Hap1^kw^ was considered to be an excellent haplotype owing to its significant increase in kernel width compared with that of Hap2^kw^ and Hap3^kw^ (Figure S7C).

Taken together, these results further suggested that natural variations in *Dek407* are associated with kernel weight and kernel size.

## 3. Discussion

### 3.1. The Function of DEK407 Differs from Its Homologs in Rice and Arabidopsis

The primary mode of nitrogen absorption in plants is through the uptake of nitrates, which are subsequently transported via nitrate transporters, including NRT1/PTR family (NPF), NRT2 family, NRT3 family, CLC family, and SLAC family [21,22]. In *Arabidopsis*, there are 53 NRT1 members, and they all belong to the NPF family [24]. These NPFs are thought to be present in all organisms, and the functions of some of them have been well described. It has been reported that the number of NPF members in rice is as many as 93 [24]. In maize, a total of 78 NPF members belonging to eight subgroups have been identified [25]. 

According to the present results, there are four homologs of DEK407 in *Arabidopsis* and rice, namely AtNPF7.3, AtNPF7.2, OsPTR2, and OsNPF7.9 (Figure 5B). With regard to OsPTR2 and OsNPF7.9, the most closely related homologs in maize were identified as Zm00001d017666 and Zm00001d051637, excluding DEK407 (Figure S5). Based on the expression data accessible in the *gramene* database (https://ensembl.gramene.org) (accessed on 1 May 2022), *OsPTR2* and *Zm00001d017666* are highly expressed in the embryo, *OsNPF7.9* and *Zm00001d051637* exhibit high expression levels in the leaf, and only *Dek407* is highly expressed exclusively in developing seeds. These results indicate that DEK407 exhibits functional differentiation from its two homologs in rice, although these proteins share a high amino acid sequence similarity. AtNPF7.3 was identified as the most closely related homolog of DEK407 in *Arabidopsis*. AtNPF7.3 is a low-affinity, pH-dependent, bidirectional nitrate transporter and is expressed in root pericycle cells close to the xylem. AtNPF7.3 participates in root xylem loading of nitrate by influencing the amount of nitrate transported from the root to the shoot, with no reported function in seed development [32]. This finding suggests that the function of DEK407 also differs from that of its homolog in *Arabidopsis*. The function of DEK407 could be special for seed development.

### 3.2. Reduction in Auxin in the dek407 Mutant May Be a New Cause for the Defective Kernel Phenotype

Auxin homeostasis plays a crucial role in the endosperm development of cereal crops. The processes of IAA biosynthesis, conjugation, oxidation, and transport can affect auxin homeostasis [49]. In rice, the expression levels of the IAA biosynthesis-related genes *OsYUC1*, *OsYUC9*, and *OsYUC11* gradually increase during development of the endosperm, which is coupled with an increase in the IAA content in the grain [50]. Mutations in both *OsYUC9* and *OsYUC11* can result in defective grain filling [51]. IAA is the most abundant endogenous hormone in maize kernels, and it plays an important role in the entire process of kernel development. Mutation of *ZmYUC1* leads to a decrease in the IAA content, abnormal differentiation of the BETL, defective endosperm filling, and a reduction in kernel size [52]. *ZmEHD1* encodes a C-terminal Eps15 homology domain (EHD) protein, and it regulates auxin homeostasis by mediating clathrin-mediated endocytosis through its interaction with the ZmAP2s subunit [53]. In kernels of the *ehd1* mutant, the content of IAA is significantly reduced compared with that of WTs, ultimately resulting in defective kernel development and vegetative growth [53].

In the transcriptome analysis of the *dek407* mutant, DEGs associated with hormone responses were identified among the significantly enriched GO categories (Figure 6B). In addition, the plant hormone signal transduction pathway was also identified as a significantly enriched KEGG pathway (Figure 6C). A total of 34 DEGs were involved in IAA synthesis and signaling in the *dek407* mutant (Figure 7A). However, the endogenous IAA content in the *dek407* mutant was only 34% of that of the WT (Figure 8A). Thus, we predicted that the significant reduction in IAA synthesis in the *dek407* mutant may be a new contributor to the defective kernel phenotype.

### 3.3. The Amino Acid Residues in Different TMs May Confer Their Specificity for Various Substrate Transportation

Compared with animals, higher plants possess a greater number of *NPF* genes. These NPF proteins are capable of transporting a variety of substrates, ions including Cl^−^ and K^+^, amino acids, sucrose, glucose, and hormones including IBA, IAA, ABA, JA, and GA [32,33,34,35]. However, the specific amino acid residues and domains of these NPFs that determine the specific binding of these different substrates are still unknown. 

In *Arabidopsis*, AtNPF7.3 is a low-affinity, pH-dependent, bidirectional nitrate transporter [38]. A recent study reported that the AtNPF7.3 protein could also function as a transporter of IBA, a precursor of the endogenous auxin [34]. When expressed in yeast, AtNPF7.3 mediated cellular uptake of IBA. The loss-of-function mutant of AtNPF7.3 showed defective root gravitropism with reduced IBA contents and auxin responses. Nevertheless, the phenotype was restored through exogenous application of IAA but not through exogenous IBA treatment [34]. As the homologous protein of AtNPF7.3 in maize, ZmSUGCAR1 has been proposed to have the capacity to transport sucrose and glucose but not IAA. The premature stop mutation in its TM9 affects sugar accumulation in the endosperm of *zmsugcar1-1* [35]. Our results showed that the G/A mutation in the third transmembrane domain (TM3) of ZmSUGCAR1 altered the expression of phytohormone-related genes and the phytohormone balance, especially the expression of IAA-related genes and the content of IAA. Then, it can be inferred that the TM3 of ZmSUGCAR1 likely plays a crucial role in facilitating IBA or IAA transport and maintaining intracellular hormone homeostasis in developing kernels.

Multidrug resistance protein 1 (MRP1) belongs to the ABC transporter superfamily. Mutagenesis studies of MRP1 have confirmed the importance of residues in different TMs in determining substrate specificity and overall activity [54]. As one of the typical members of the RND (resistance–nodulation–division) superfamily, the *Escherichia coli* OqxAB multidrug efflux pump confers resistance to antimicrobial agents, such as olaquindox and fluoroquinolone. A molecular docking analysis demonstrated that residues from different domains of OqxAB exhibit diverse functions in the transportation of various substrates [55]. In the *zmsugcar1-1* mutant, the premature stop mutation in the TM9 of ZmSUGCAR1 resulted in the loss of the last three TMs but had no effect on the other TMs [35]. In the *dek407* mutant, the G/A mutation in TM3 of ZmSUGCAR1 leads to an alteration of the corresponding coding amino acid from Cys to Tyr, subsequently altering the expression of phytohormone-related genes and the phytohormone balance, especially the expression of IAA-related genes and the content of IAA. This new finding, which differs from *zmsugcar1-1*, suggests that different amino acid residues or different TMs of ZmSUGCAR1 may be responsible for the specific recognition and interaction with different substrates. Whether ZmSUGCAR1 could transport IAA or its precursor IBA and cooperate with other proteins in vivo through specific amino acid residues remains unknown and is an attractive possibility. This intriguing hypothesis requires further investigation in future studies.

### 3.4. Dek407 Is a Potential Target for the Improvement of Maize Yield

The domestication of maize can be traced to approximately 9000 years ago in Southern Mexico. Through a long process of selection and domestication, the progenitor teosinte has been transformed into today’s high-yielding, high-quality, and stress-resistant maize varieties [56]. During this process, variations in the gene loci that contribute to yield and stress resistance were selected by breeders, and the frequency of these elite allelic variants were continuously enriched. A recent study has shown that natural variation in *ZmUrb2* is associated with maize kernel size, and that inbred lines with haplotype 3 have significantly longer kernels than those with other haplotypes [15]. These identified loci or genes can serve as direct targets of genetic selection for maize trait improvement. In the present study, we determined that maize inbred lines with Hap1^hkw^ of *Dek407* had a significantly higher hundred-kernel weight than those lines with Hap2^hkw^, Hap3^hkw^, Hap4^hkw^, and Hap5^hkw^. Thus, we infer that an elite haplotype of *Dek407* may be helpful to increase maize yield. Therefore, *Dek407* is a potential target for the improvement of maize yield.

## 4. Materials and Methods

### 4.1. Plant Materials

The maize (*Zea mays* L.) mutant *dek407* was isolated by screening the EMS mutagenesis library of the B73 inbred line [43]. The *dek407* mutant was crossed with the Zheng58 and C01 genetic backgrounds to generate F_2_ populations for genetic analysis and gene mapping. All the plants were grown in the experimental field of Henan Agricultural University under natural conditions (Zhengzhou, China).

### 4.2. Light Microscopy of Cytological Sections

Wild-type and *dek407* mutant kernels from the same heterozygous ear at 12 DAP were collected for light microscopy visualization of cytological sections. To prepare the paraffin sections of the kernels, 12 DAP seeds were fixed overnight at 4 °C in a formalin-acetic acid–alcohol (FAA) solution. The fixed materials were then dehydrated in an ethanol gradient series of 70%, 75%, 80%, 85%, 90%, 95%, and 100% ethanol for 2 h at each step. Afterwards, the samples were treated with xylene, embedded in paraffin wax via infiltration, and cut into 10–12 µm thick sections using a microtome (Leica RM2235, Heidelberg, Germany). The sections were stained with toluidine blue (Sinopharm Chemical Reagent Co., Ltd. Shanghai, China) and examined under a Leica M165FC stereomicroscope (Leica M165FC, Heidelberg, Germany). 

### 4.3. Measurement of Hundred-Kernel Weight and Starch Content

The self-pollinated heterozygous ears were harvested from the field to isolate wild-type and mutant (*dek407*, *dek407-1*, and *dek407-2*) kernels and dried to a constant weight. Three ears were utilized as individual biological replicates. The weight of one hundred WT and mutant kernels from each ear was measured using a laboratory scale. For starch content analysis, the WT and *dek407* seeds harvested at 45 DAP were dried at 80 °C to a constant weight and subsequently pulverized into a fine powder using a pestle and mortar. For each sample, 50 mg of flour was used to measure the starch content using a starch content assay kit (Beijing Solarbio Science & Technology Co., Ltd. BC0700, Beijing, China). All the measurements were conducted on three biological replicates.

### 4.4. Bulked Segregant RNA-Seq Analysis

The *dek407* mutant was crossed with C01, and self-pollinated F_2_ populations were used for BSR-seq. WT and mutant kernels were collected from the same ear at 15 DAP. The pericarps were removed, and 30 kernels of either the WT or mutant phenotype were pooled for the BSR analysis. The total RNA was extracted from each pool using TransZol Plant (TransGen, ET121-01, Beijing, China) and subjected to Illumina sequencing in Hiseq2000 (Berry Genomics, Fuzhou, China). A BSR-seq analysis was performed following the method of Liu et al. [57]. In brief, the sequences were mapped against the B73 genome (AGPv4.32). Single-nucleotide polymorphism (SNP) callings were processed using SAMtools (https://www.htslib.org/ accessed on 1 May 2022). High-quality SNPs were selected for the SNP index analysis. Δ (SNP-index) was calculated by subtracting the SNP index of the mutant pool from the WT pool. A graph of the average values of Δ (SNP-index) was plotted using a 1 Mb window size and a 1 kb window step size.

### 4.5. Map-Based Cloning of Dek407

The F_2_ mapping population was derived from a cross between *dek407* and the maize inbred line C01. Map-based cloning was performed using 288 mutant individuals from the F_2_ mapping population. For fine mapping, InDel and SSR molecular markers (Table S4) were developed to narrow the *Dek407* locus to a 5.9 Mb region. To identify the mutation site, the candidate genes were amplified from the mutant and WT kernels using Phanta Max Super-Fidelidy DNA polymerase (Vazyme, P505, Nanjing, China) and were subsequently sequenced.

### 4.6. Sequence BLAST and Phylogenetic Tree Construction

The DEK407 protein was used as the query for BLAST searches against the *Zea mays*, *Sorghum bicolor*, *Setaria italica*, *Oryza sativa*, *Arabidopsis thaliana*, *Glycine max*, *Hordeum vulgare*, *Prunus persica*, *Populus tremula*, *Poncirus trifoliata*, and *Paspalum vaginatum* genomes on phytozome (https://phytozome.jgi.doe.gov) (accessed on 1 May 2022). A multiple sequence alignment was performed using the protein sequences encoded by 30 and 50 well-known *NRT1*/*PTR* family (*NPF*) genes in rice and *Arabidopsis thaliana* and their homologous in maize. These protein sequences were aligned using the MUSCLE algorithm implemented in MEGA 11 (https://www.megasoftware.net/ accessed on 1 May 2022). The phylogenetic tree was subsequently constructed based on the alignment results using the neighbor-joining (NJ) method implemented in MEGA 11. Bootstrapping was performed with 1000 replications.

### 4.7. RNA-Sequencing Analysis

The total RNA of three biological replicates was extracted from 15 DAP WT and *dek407* kernels with the pericarp removed using TransZol Plant (TransGen, ET121-01, Beijing, China). Clean reads were obtained using the Illumina HiSeq X Ten platform (Berry Genomics, Fuzhou, China) and mapped to the B73 reference genome (RefGen_V4). The gene expression value was normalized as fragments per kilobase of transcript per million mapped reads (FPKM). The DEGs were produced using the threshold of a *p*-value < 0.05 in the DESeq software package (http://bioconductor.org/ accessed on 1 May 2022). GO enrichment of DEGs was performed using the DESeq R package based on the hypergeometric distribution. 

### 4.8. Measurement of Endogenous Phytohormones

Approximately 2 g of WT and *dek407* kernels was ground into fine powder in the presence of liquid nitrogen for the measurement of endogenous phytohormones. According to the methods of Xin et al., the powder was extracted using 1.0 mL of 80% methanol (*v*/*v*) at 4 °C for 12 h [58]. The supernatant was obtained after centrifugation at 10,000× *g* for 20 min at 4 °C. The solvent was evaporated under a gentle nitrogen stream at 35 °C and then re-dissolved in 40% methanol with a volume of 100 μL. The filtrate was filtered through a 0.22 µm filter membrane after shock mixing. Subsequently, the phytohormones were quantified using ultra-high-performance liquid chromatography–tandem mass spectrometry (UPLC-MS/MS) using a Xevo TQ-XS system (Waters, Milford, MA, USA). The flow rate of the mobile phase, composed of solvent A (0.1% formic acid, water) and solvent B (methanol), was set to 0.3 mL/min. The linear-gradient system was set as follows: 0–2 min, 2% B; 2–10 min, up to 80% B; 10–12 min, 80% B; 12–13 min, down to 2% B; 13–15 min, 2% B. The autosampler temperature was set to 4 °C, and the sample injection volume was 10 μL. Three biological replicates were prepared for each measurement.

### 4.9. Candidate Gene-Based Association Analysis

A total of 503 maize inbred lines were utilized for the candidate gene-based association analysis, with corresponding genotyping data of *Dek407* and phenotypic data acquired from MaizeGo (http://www.maizego.org/Resources.html) (accessed on 1 May 2022). The SNP data were filtered based on a minor allele frequency (MAF) of >0.05 and a missing rate of <20%. The candidate gene-based association analysis was conducted using TASSEL 5.0. A linkage disequilibrium (LD) plot was generated using HAPLOVIEW software (https://sourceforge.net/projects/haploview/) (accessed on 1 May 2022). Differences in the phenotypic values and haplotypes were analyzed using a one-way ANOVA or Student’s *t*-tests.

## Data Availability

The data presented in this study are available in this article and the Supplementary Materials.

## Supplementary Materials

The following supporting information can be downloaded at: https://www.mdpi.com/article/10.3390/ijms242417471/s1.
