# Supplementary figures and images for: Maize Dek407 Encodes the Nitrate Transporter 1.5 and Is Required for Kernel Development
Source: Int J Mol Sci. 2023 Dec 14;24(24):17471. doi: 10.3390/ijms242417471 (PMC10743814; doi:10.3390/ijms242417471)

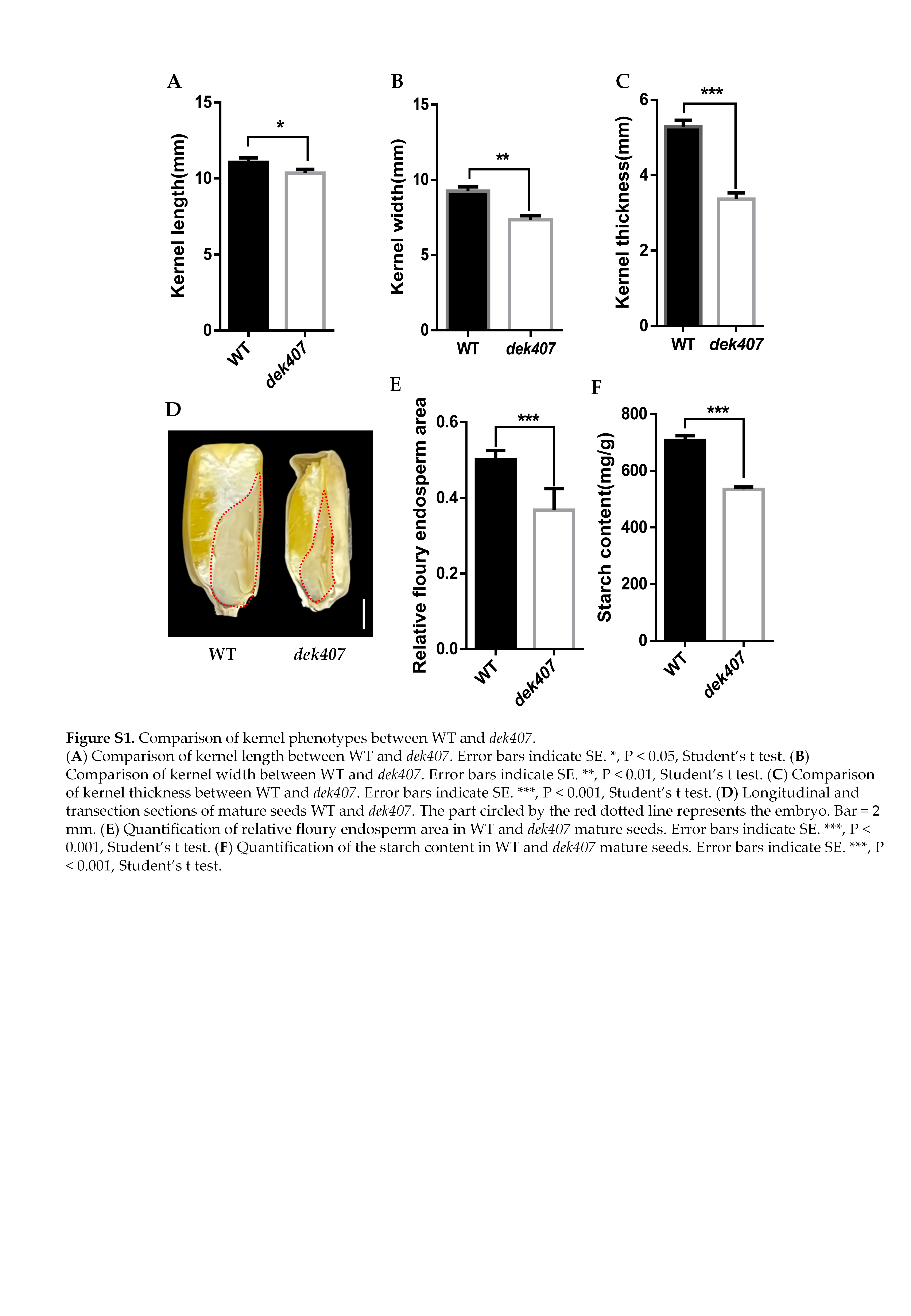

Supplement: Supplementary file 1 [file ijms-24-17471-s001.zip › Supplementary Figures_Figure S1.png]

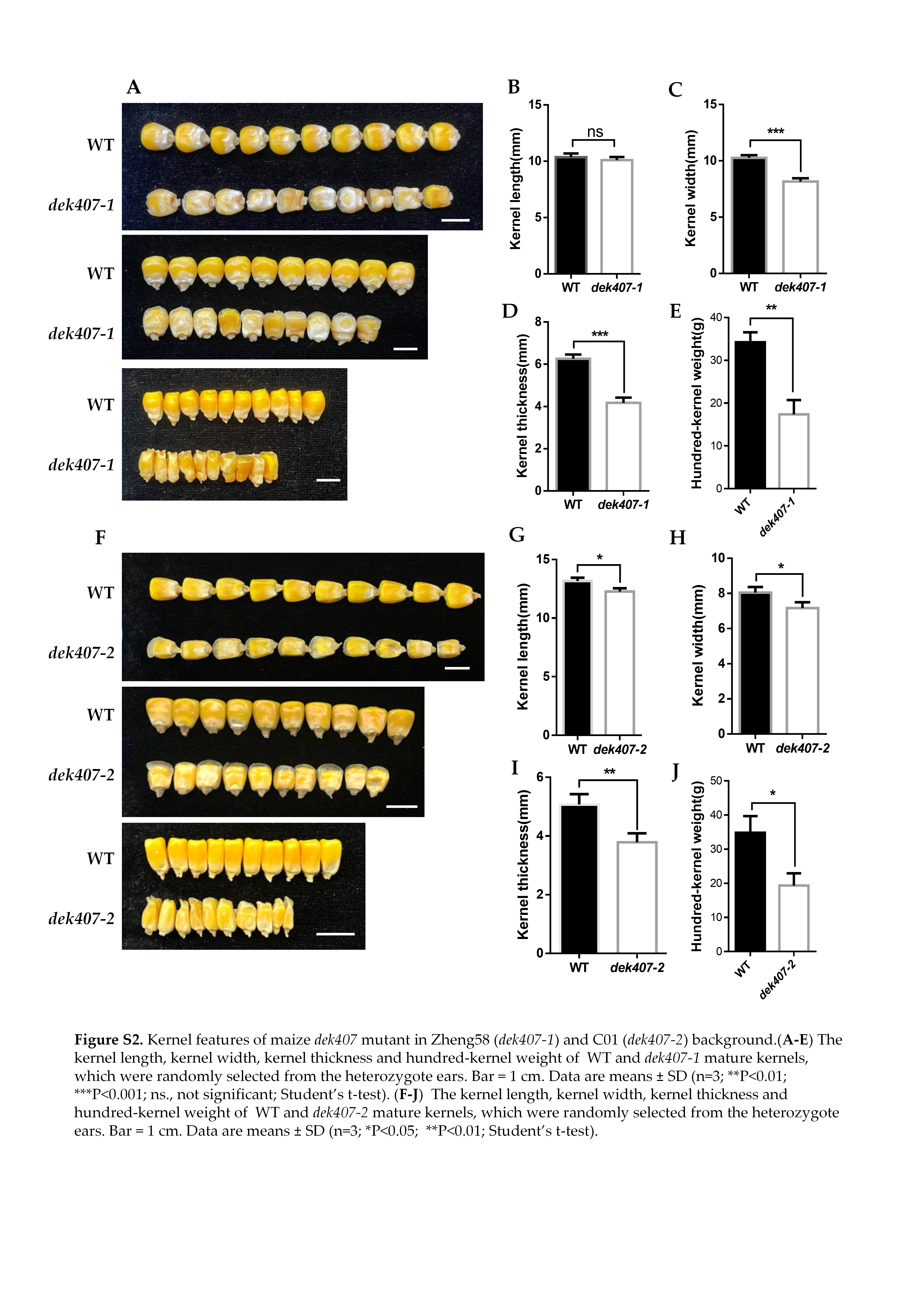

Supplement: Supplementary file 1 [file ijms-24-17471-s001.zip › Supplementary Figures_Figure S2.png]

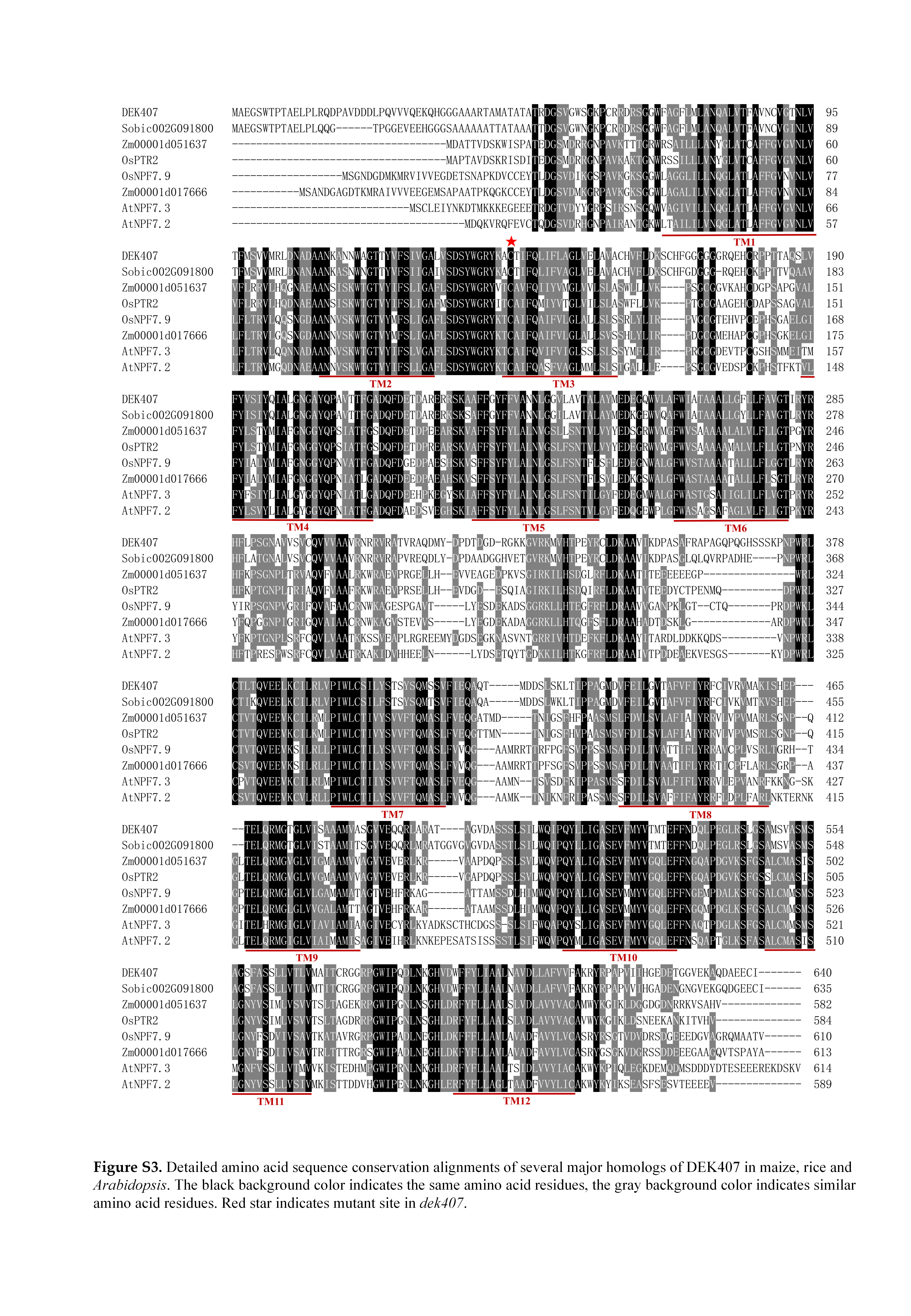

Supplement: Supplementary file 1 [file ijms-24-17471-s001.zip › Supplementary Figures_Figure S3.png]

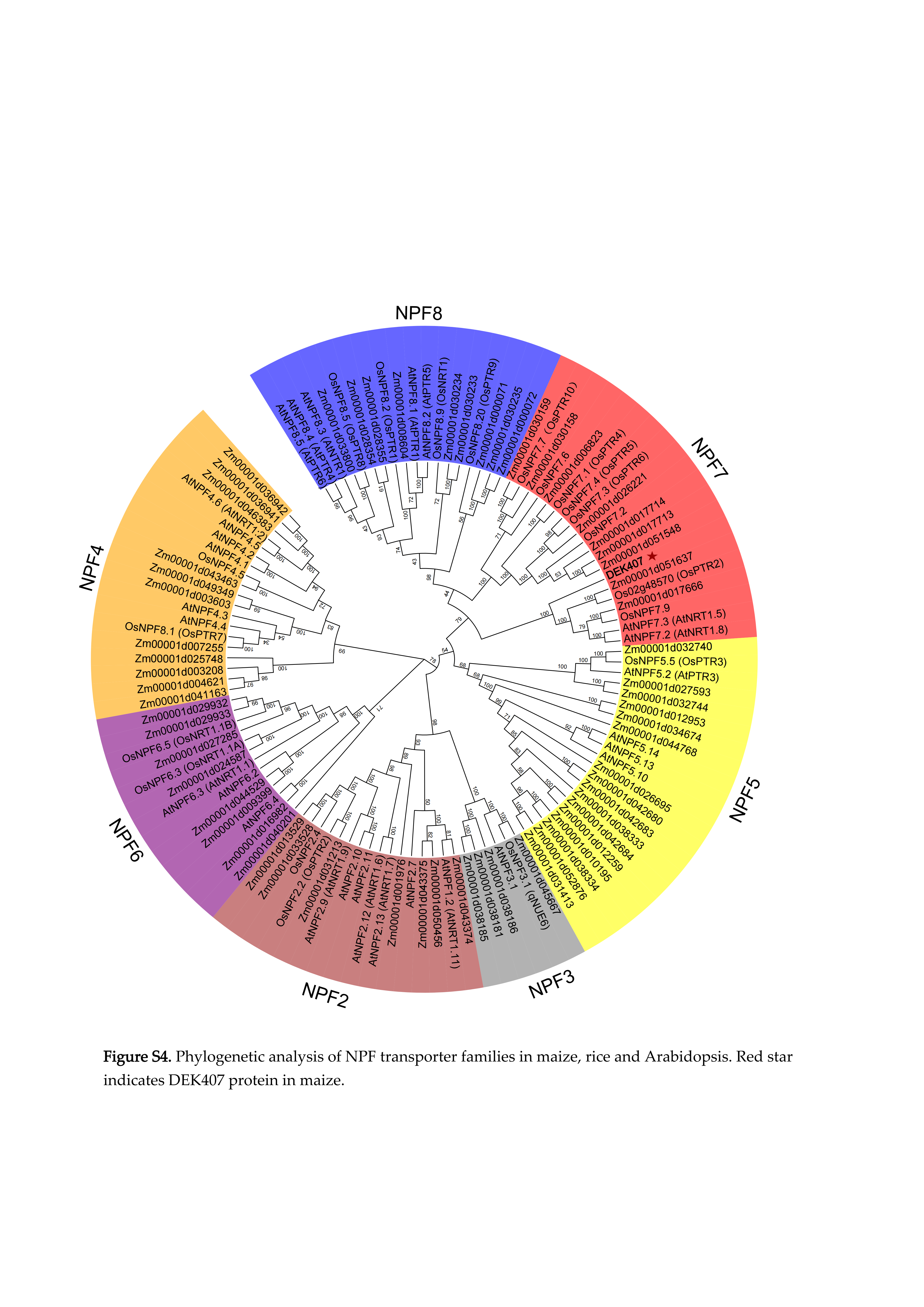

Supplement: Supplementary file 1 [file ijms-24-17471-s001.zip › Supplementary Figures_Figure S4.png]

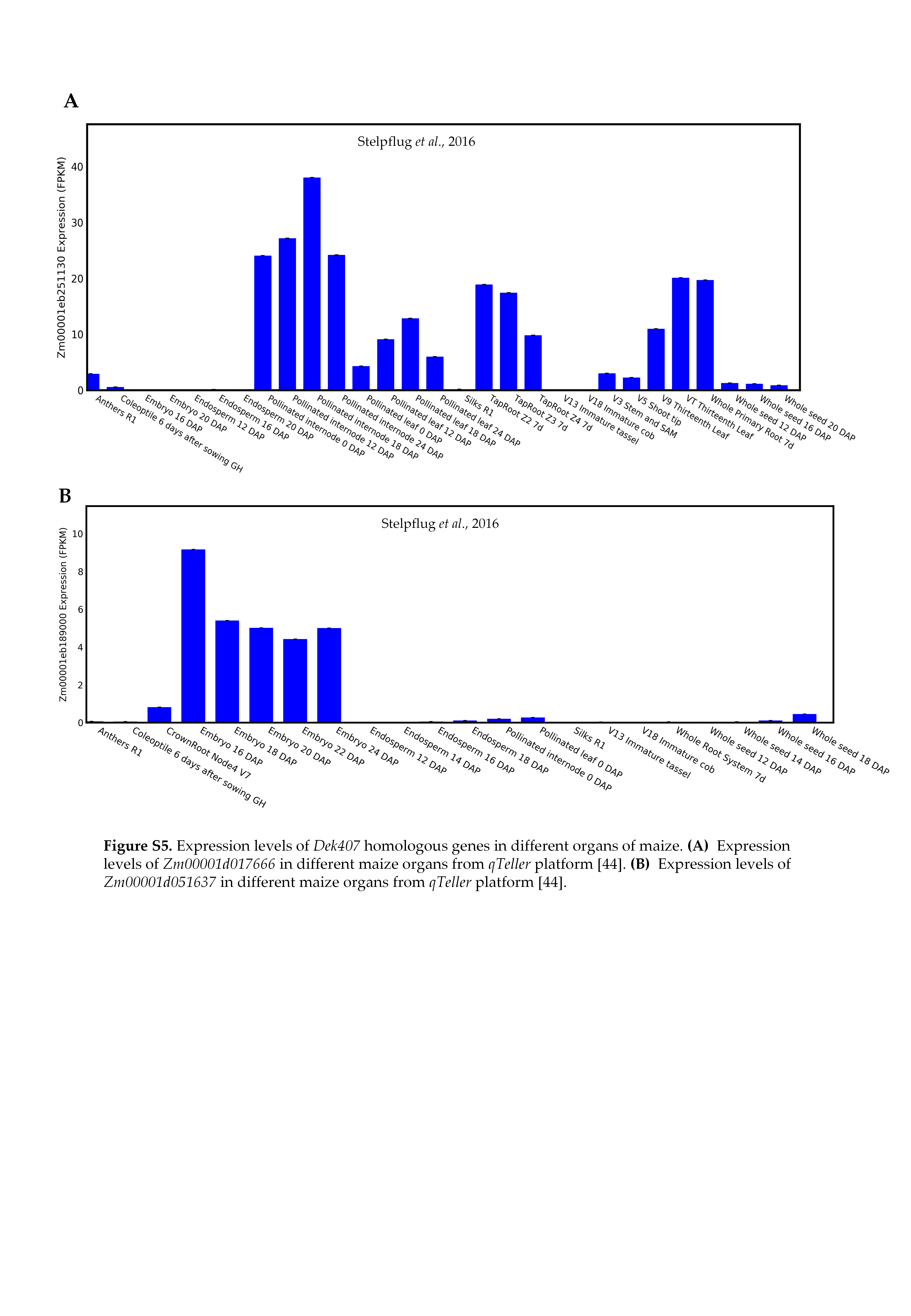

Supplement: Supplementary file 1 [file ijms-24-17471-s001.zip › Supplementary Figures_Figure S5.png]

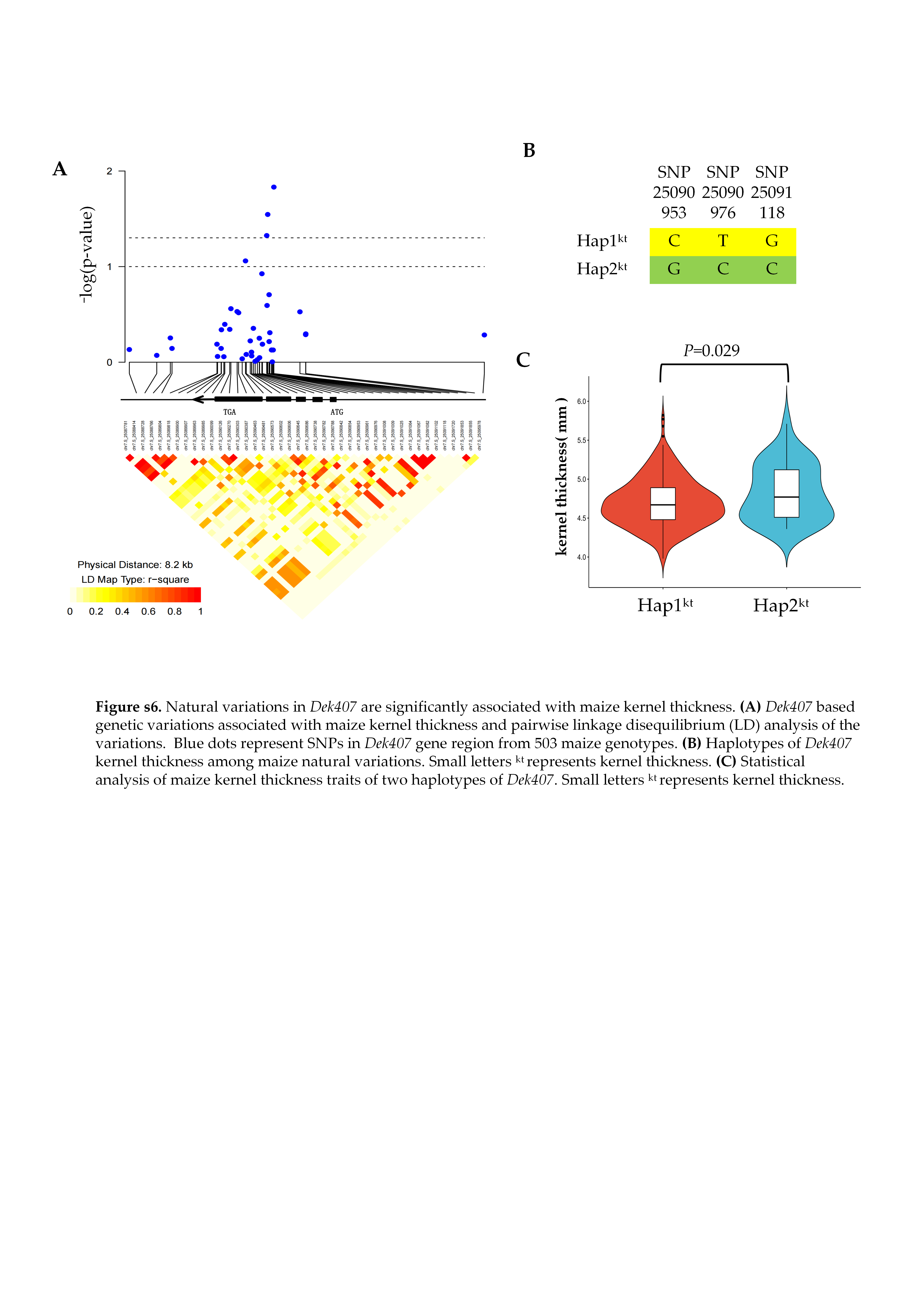

Supplement: Supplementary file 1 [file ijms-24-17471-s001.zip › Supplementary Figures_Figure S6.png]

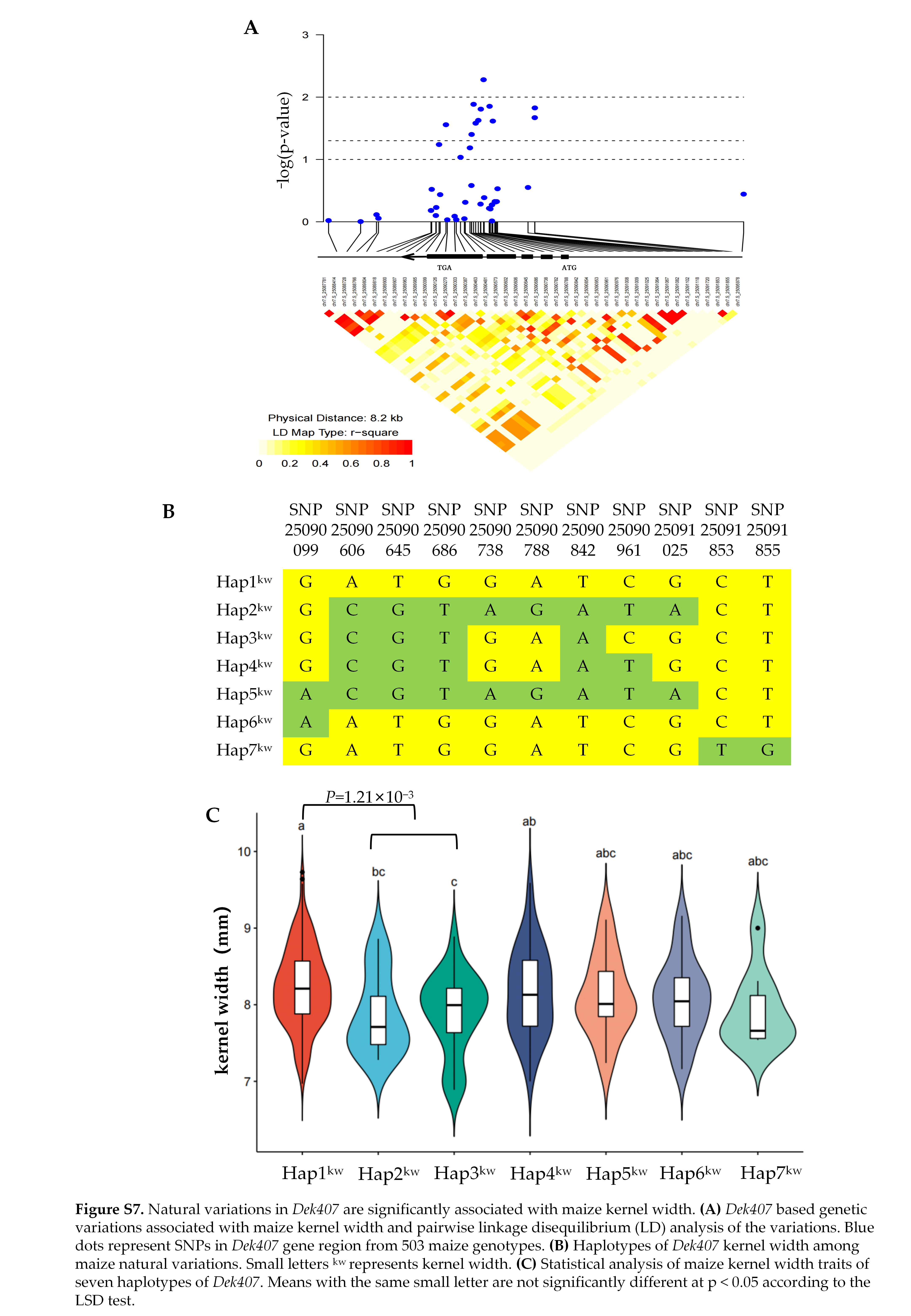

Supplement: Supplementary file 1 [file ijms-24-17471-s001.zip › Supplementary Figures_Figure S7.png]
